# Comparative study of phenotypic-based detection assays for carbapenemase-producing *Acinetobacter baumannii* with a proposed algorithm in resource-limited settings

**DOI:** 10.1371/journal.pone.0259686

**Published:** 2021-11-04

**Authors:** Piyatip Khuntayaporn, Krit Thirapanmethee, Pohnvipa Kanathum, Kanokwan Chitsombat, Mullika Traidej Chomnawang

**Affiliations:** 1 Department of Microbiology, Faculty of Pharmacy, Mahidol University, Bangkok, Thailand; 2 The Antimicrobial Resistance Interdisciplinary Group (AmRIG), Faculty of Pharmacy, Mahidol University, Bangkok, Thailand; 3 Biopharmaceutical Sciences Program, Faculty of Pharmacy, Mahidol University, Bangkok, Thailand; Nitte University, INDIA

## Abstract

The increasing incidence of carbapenem resistance in *Acinetobacter baumannii* is a critical concern worldwide owing to the limitations of therapeutic alternatives. The most important carbapenem resistance mechanism for *A*. *baumannii* is the enzymatic hydrolysis mediated by carbapenemases, mostly OXA-type carbapenemases (class D) and, to a lesser extent, metallo-β-lactamases (class B). Therefore, early and accurate detection of carbapenemase-producing *A*. *baumannii* is required to achieve the therapeutic efficacy of such infections. Many methods for carbapenemase detection have been proposed as effective tests for *A*. *baumannii*; however, none of them are officially recommended. In this study, three carbapenemase detection methods, namely, CarbaAcineto NP test, modified carbapenem inactivation method (mCIM), and simplified carbapenem inactivation method (sCIM) were evaluated for phenotypic detection of clinically isolated *A*. *baumannii*. The MICs of imipenem, meropenem, and doripenem were determined for 123 clinically isolated *A*. *baumannii* strains before performing three phenotypic detections. The overall sensitivity and specificity values were 89.09%/100% for the carbAcineto NP test, 71.82%/100% for sCIM, and 32.73%/33.13% for mCIM. CarbAcineto NP test and sCIM performed excellently (100% sensitivity) when both Class B and Class D carbapenemases were present in the same isolate. Based on the results, the combined detection method of sCIM and CarbAcineto NP test was proposed to detect carbapenemase-producing *A*. *baumannii* rather than a single assay, significantly increasing the sensitivity of detection to 98.18%. The proposed algorithm was more reliable and cost-effective than the CarbAcineto NP test alone. It can be easily applied in routine microbiology laboratories for developing countries with limited resources.

## Introduction

Increasing antibiotic resistance has raised awareness of the healthcare system worldwide. The beta-lactam drug group, which can be categorized into four major drug groups: penicillins, cephalosporins, monobactams, and carbapenems, accounts for half of the currently used antibiotics [1]. Carbapenems are considered the last resort drugs for severe infections because of their clinical efficacy and enzyme resistance activities. However, bacteria can mutate and escape from beta-lactam drugs by beta-lactamase production, the most prevalent resistance mechanism against beta-lactams in gram-negative bacteria. Although most beta-lactamases cannot hydrolyze carbapenems [2], beta-lactamases capable of destroying carbapenems have been discovered since the 1990s and are a health threat [3].

*Acinetobacter baumannii* is an opportunistic pathogen that frequently involves nosocomial infections, including lower respiratory tract infections, urinary tract infections, wound infections, and bacteremia, particularly in intensive care unit patients [4]. The Center for Disease Control and Prevention (CDC) and World Health Organization (WHO) defined carbapenem-resistant *A*. *baumannii* (CR-AB) as one of the most urgent health threats in this century [5, 6]. This organism can be resistant to the most currently available antibiotics. According to the surveillance data in Thailand by the National Antimicrobial Resistance Surveillance Center, Thailand (NARST), imipenem resistance in *Acinetobacter* spp. has been reported to be over 50% since 2007 [7]. The most common beta-lactamases found in *A*. *baumannii* are OXA-type carbapenemases, which belong to class D carbapenemases [8]. OXA-51-like is an intrinsic chromosomal carbapenemase in *A*. *baumannii*, whereas OXA-23-like, 24/40-like, and -58-like are acquired carbapenemases frequently found in *A*. *baumannii* [8, 9]. Among these, OXA-23 has been reported globally and is associated with carbapenem resistance [10]. In Thailand, OXA-23-like was also identified as a predominant OXA-carbapenemase in *A*. *baumannii* [11].

The European Committee on Antimicrobial Susceptibility Testing (EUCAST) and the Clinical and Laboratory Standards Institute (CLSI) issued guidelines on carbapenemase detection for Enterobacterales and *Pseudomonas aeruginosa* [12, 13]. There is no suggestion to identify resistance mechanisms, and lower breakpoints should be sufficient for treatment purposes [14]. However, determination of resistance mechanisms is still necessary for effective treatment, particularly for transferable resistance mechanisms, such as antibiotic resistance genes found on plasmids or transposons [10]. In epidemiology, the early detection of transferable beta-lactamase enzymes, particularly transferable carbapenemases, is crucial for infection control and local outbreak monitoring. A method with reliability and rapidity to detect resistance mechanisms has become essential.

There are many available detection methods for carbapenemases in Enterobacterales and *Pseudomonas* spp., such as the paper-based diffusion method, carbapenemase Nordmann-Poirel (Carba NP) test, and matrix-assisted laser desorption ionization-time of flight mass spectrometry (MALDI-TOF) [12, 13]. However, there is no recommended method for carbapenemase detection in *Acinetobacter* spp. Several phenotypic detection methods have been proposed, such as MALDI-TOF, CarbAcineto NP test, and the paper-based diffusion method. The principle of MALDI-TOF for carbapenemase detection is to determine the imipenem hydrolysis of selected bacterial isolates [15]. This method is rapid and has high sensitivity and specificity; however, it requires very sophisticated instruments and specialists with specific training. Moreover, the machine is costly to maintain and would be available only in tertiary or quaternary hospitals in low-to middle-income countries.

The Carba NP test was recommended in the CLSI guideline as a carbapenemase detection method for Enterobacterales and *Pseudomonas aeruginosa* since 2015, not long after publication [16]. However, in the case of *Acinetobacter* spp., the reliability of the Carba NP test was not sufficient when compared to that for the bacteria mentioned above. Therefore, the CarbAcineto NP test was developed in 2014, modified from the Carba NP test in terms of lysis conditions and bacterial inoculum amount [17]. The principle of this method depends on the change in color of the pH indicator, phenol red, due to the shifting of pH in the tested solution after incubation of imipenem with a tested bacterial isolate. This method is fast, user-friendly, and does not require specific instruments. However, the interpretation is quite subjective, based on observations of the color change, and the test kit is still high-priced for routine laboratory surveillance, particularly in limited resource settings. In addition, inconsistencies were observed with the ability to detect OXA-51-like carbapenemases.

The paper-based diffusion method is the simplest and easiest to apply in most laboratories for routine microbiological work; however, the criteria guideline of this method needs validation before application in clinical settings, particularly in difficult-to-detect bacteria such as *Acinetobacter* spp. The most recent paper-based carbapenemase detection in the CLSI guideline is the modified carbapenem inactivation method (mCIM) and EDTA-modified carbapenem inactivation method (eCIM) [18]. Both methods determine the diameter of the carbapenem inhibition zone and the growth of bacteria inside the inhibition zone after incubation with an antibiotic disk with suspected carbapenemase-producing strains. These methods are not recommended for *A*. *baumannii* infections. Later, the sCIM was developed in 2018 to simplify the working procedure and enhance the specificity and sensitivity of *A*. *baumannii* [19]. However, all carbapenemase detection methods still require validation before application in clinical settings for *A*. *baumannii*. Therefore, in this study, three phenotype-based detection methods were investigated for clinically isolated *A*. *baumannii*. In addition, the test performance was evaluated to detect the most common beta-lactamase types found in *A*. *baumannii*. Based on the results, a new algorithm for carbapenemase detection in *A*. *baumannii* was proposed, considering the limitations of low-to middle–income countries.

## Materials and methods

### Study setting and collection

A total of 123 *A*. *baumannii* isolates were collected from adult patients admitted to 11 tertiary care hospitals in Thailand during 2016–2017. Non-duplicate consecutive isolates from distinct infectious episodes were obtained from admitted patients. All isolates were obtained from clinical samples (sputum, urine, pus, tissue, or blood) from non-duplicate patients. Geographically, all isolates were collected from three hospitals in the central region, three hospitals in the northern region, three hospitals in the eastern region, and two hospitals in the southern region. The study was approved by the Ethical Review Committee of the Faculty of Dentistry and Faculty of Pharmacy, Mahidol University (MU-DT/PY-IRB 2016/008.0404).

### Bacterial identification

Acinetobacter isolates were identified using microbiological and biochemical methods. The identity was confirmed by the molecular method, namely multi-locus sequence typing analysis (MLST) according to the Institute Pasteur protocol. The primers specific for seven housekeeping genes (*recA*, *gltA*, *fusA*, *cpn60*, *pyrG*, *rplB*, and *rpoB*) were used for PCR amplification, and PCR products were purified using a commercial kit according to the manufacturer’s instructions (Favorgen, Ping-Tung, Taiwan). Nucleotide sequencing was performed by Bio Basic Asia Pacific Pte Ltd., Singapore. The nucleotide sequences were analyzed as previously described by comparing them to those in the Institute Pasteur database (https://pubmlst.org/abaumannii/) [20].

### Antimicrobial susceptibility test

Antimicrobial susceptibility testing for CR-AB identification was performed using broth microdilution methods according to the Clinical Laboratory Standard Institute (CLSI) guidelines [21]. *A*. *baumannii* isolates were grown in cation-adjusted Mueller-Hinton broth (CAMHB, Difco, BD Diagnostic Systems, USA) before adding them into 96-well plates containing antibiotics to the final concentration (approximately 10^5^ CFU/ml). *A*. *baumannii* isolates were tested against carbapenems, including imipenem, meropenem, and doripenem (Carbosynth, UK). The microtiter plate was incubated for 16–20 h at 35±2°C. The results were evaluated based on the MIC values from the minimum concentration of drugs that showed no visible growth. Clinical and Laboratory Standards Institute (CLSI) breakpoints were used for susceptibility determination. In this study, CR-AB was defined as an isolate resistant to at least one carbapenem agent. *A*. *baumannii* ATCC 19606, obtained from the American Type Culture Collection (ATCC, Manassas, VA, USA) was used as a quality control isolate.

### CarbAcineto NP test

CarbAcineto NP staining was performed as previously described [17]. *A*. *baumannii* clinical isolates were cultured overnight in TSA. Each tested isolate was prepared in two 1.5 ml microcentrifuge tubes (Tube A and B). One full loop of tested bacteria was harvested and resuspended by vortexing for 15 s at full speed in 100 μL of lysis buffer, which was prepared from a 5 M NaCl solution. The revealing solution containing phenol red was added to 100 μL in tube A, whereas the same solution with an additional 6 mg/ml imipenem was added to tube B. Both tubes were incubated at 35±2°C for 2 h. The color change of both tubes was observed by the naked eye. The color change only in tube B was interpreted as a positive result, whereas both tubes remaining red were recorded as negative results.

### Modified carbapenem inactivation method (mCIM)

mCIM was performed as previously recommended [22]. A 10 μl loopful of *A*. *baumannii* was added to 2 ml tryptic soy broth (TSB) and the mixture was mixed vigorously for 15 s. Thereafter, 10 μg meropenem (MEM) disk was added to the bacterial suspension and incubated at 35±2°C for 4 h. *Escherichia coli* ATCC25922, with turbidity equivalent to 0.5, was swabbed on Mueller-Hinton agar (MHA) shortly before disk incubation. Furthermore, the MEM disk was removed from the bacterial suspension and placed onto the inoculated MHA. The MHA plate was incubated overnight at 35±2°C, and the inhibition zone diameter was measured. Zone diameter ≥19 mm was interpreted as negative results, inhibition zone diameter of 6–15 mm or colonies within a 16–18 mm zone were considered a positive result. Indeterminate results were interpreted when zone diameter within 16–18 mm or zone diameter ≥19 with colonies within the zone [18, 19].

### Simplified carbapenem inactivation method (sCIM)

In contrast to the other methods, sCIM was performed by directly applying the tested isolate onto one side of the antibiotic disk. The procedure was performed as described by Jing et al. [19]. Isolated cells were cultured overnight on TSA. *E*. *coli* ATCC25922 was adjusted to 0.5 McFarland and diluted 1:10 in a normal saline solution before swabbing on an MHA plate. The MHA plate was allowed to dry for 5–15 min. Thereafter, a 10-μg imipenem disk that was smeared on one side with the tested isolate was placed onto the plate. The MHA plate was incubated overnight at 35±2°C, and the inhibition zone diameter was measured. Zone diameter ≥26 mm was considered a negative result, whereas inhibition zone 6–20 mm or satellite colonies within zone diameter ≤22 mm was interpreted as a positive result. The indeterminate result was interpreted when zone diameter was within 23–25 mm.

### Genotypic determination of carbapenemases

The polymerase chain reaction (PCR) methods were performed for amplification of *bla*oxa-23-like, *bla*oxa-24/40-like, *bla*oxa-51-like, *bla*oxa-58-like, and *ndm*-type *A*. *baumannii* isolates as previously described [11]. Briefly, the amplification process for the OXA-23-like gene was initial denaturation at 94°C for 5 min, 30 cycles of 94°C for 25 s, 55°C for 40 s, and 72°C for 50 s, and a final elongation at 72°C for 6 min. The amplification process for the OXA-24/40-like and OXA-51-like genes was initial denaturation at 94°C for 5 min, 30 cycles of 94°C for 25 s, 58°C for 40 s, and 72°C for 50 s, and a final elongation at 72°C for 6 min. The amplification process for the intrinsic OXA-58-like gene was initial denaturation at 94°C for 5 min, 30 cycles of 94°C for 25 s, 64°C for 40 s, and 72°C for 50 s, and a final elongation at 72°C for 6 min. For *ndm*-type carbapenemase, the amplification process began with initial denaturation at 94°C for 3 min, 30 cycles of 94°C for 30 s, 57°C for 30 s, and 72°C for 30 s, and a final elongation at 72°C for 3 min. *E*. *coli* ATCC BAA 2469 obtained from the American Type Culture Collection, USA was used as a positive control for ndm-type carbapenemase. All CR-AB isolates were confirmed by PCR method. The isolates harboring at least one carbapenemase gene were considered as true positive isolates. The true negative isolates were carbapenem-susceptible *A*. *baumannii* isolates with no carbapenemase gene as demonstrated by the PCR method.

### Data analysis

Sensitivity, specificity, positive and negative predictive values, and confidence intervals (CIs) were calculated using the Internet-based software MedCalc® (Available from: https://www.medcalc.org/calc/diagnostic_test.php). In this study, sensitivity and specificity were calculated according to the method of Trevethan R [23]. To date, the gold standard method for genotypic detection for carbapenem resistance in *A*. *baumannii* is still the PCR method. Sensitivity is a probability of being test positive when disease present which can be calculated by true positive divided by the summation of all positive results. Specificity is a probability of being test negative when disease absent which can be calculated by true negative divided by the summation of all negative results. All indeterminate results were recognized as positive only in the combination test calculations. The prevalence used to calculate positive and negative predictive values was based on the prevalence of carbapenemase-resistant *A*. *baumannii* in Thailand, estimated to be approximately 75% [7]. The time consumption and expenses of consumable equipment were calculated based on the performance of the researcher’s laboratory.

## Results

### Comparison of the performances of carbapenemase-detection methods

A total of 123 well-characterized *A*. *baumannii* isolates were selected for this study, out of which 89.43% (110/123) isolates showed resistance to carbapenems on screening tests. Among the 110 *A*. *baumannii* isolates, 89.09% (98/110) were positive for the CarbAcineto NP test, and 10.91% (12/110) were negative. In contrast, sCIM demonstrated 71.82% positive results, and only 32.73% (36/110) showed the presence of carbapenemase activity by mCIM. The performance characteristics of all carbapenemase-detection methods are presented in Table 1. The carbapenemase-detection method that exhibited the highest sensitivity was the CarbAcineto NP test, followed by sCIM, whereas the sensitivity of mCIM was much lower. However, when combined with the positive and indeterminate results of sCIM, the sensitivity was equivalent to the CarbAcineto NP test. When considering the specificity of target carbapenemases, CarbAcineto NP test and sCIM exhibited 100% specificity in detecting carbapenemase-producing strains, which was incomparable to mCIM (33.13%). The overall accuracy of all detection methods could be ranked from the CarbAcineto NP test, sCIM, and mCIM with a significant difference.

**Table 1:** Performance characteristics of phenotypic methods for detection of carbapenemase-producing *A*. *baumannii*.

Clinical *A*. *baumannii* isolates	CarbAcineto NP (n)			mCIM			sCIM		
				(n)			(n)		
	P	I	N	P	I	N	P	I	N
**Carbapenem resistant**	98	0	12	36	0	74	79	19	12
(Carbapenemase producers)									
n = 110									
**Carbapenem sensitive**	0	0	13	0	1	12	0	0	13
(Non-carbapenemase producers)									
n = 13									
**Sensitivity,** % (95% CI)	89.09%			32.73%			71.82%		
	(81.72–94.23%)			(24.08–42.33%)			(62.44–79.98%)		
**Specificity,** % (95% CI)	100.00%			33.13%			100.00%		
	(75.29–100.00%)			(30.31–36.08%)			(75.29–100.00%)		
**Positive Predictive Value,** % (95% CI)	100%			100%			100%		
**Negative predictive Value,** % (95% CI)	75.34%			33.13%			54.19%		
	(64.17–83.90%)			(30.31–36.08%)			(46.74–61.45%)		
**Accuracy,** % (95% CI)	91.82%			49.55%			78.86%		
	(85.49–96.00%)			(40.41–58.70%)			(70.58–85.70%)		

P, positive; I, Indeterminate; N, Negative; n, number of isolates

### The detection test performance according to the genotypic beta-lactamase types found in *A*. *baumannii*

Further studies on the performance of carbapenemase-detection methods according to beta-lactamase types commonly found in *A*. *baumannii* are shown in Table 2. CR-AB isolates were selected and genotypically characterized according to the epidemiology of *A*. *baumannii* in Thailand. Among all assays, the CarbAcineto NP test exhibited the highest sensitivity in detecting all class D carbapenemases, a single enzyme type and co-existence strains. Similar results were obtained for sCIM, except for OXA-24 alone or in combination with OXA-58. In addition, mCIM had much lower sensitivity in identifying all class D carbapenemases, particularly OXA-23 (29.67%). CarbAcineto NP test and sCIM performed excellently (100% sensitivity) when both Class B and Class D carbapenemases were present in the co-producer isolate.

**Table 2:** Performance of phenotypic detection methods according to genotypic characterization for *A*. *baumannii*.

Clinical	CarbAcineto NP			mCIM			sCIM			MIC range		
*A*. *baumannii* isolates	n (%)			n (%)			n (%)			(μg/ml)		
	P	I	N	P	I	N	P	I	N	Imipenem	Meropenem	Doripenem
**Class D carbapenemases**												
OXA-23	81 (89.01%)	0	10 (10.99%)	27 (29.67%)	0	64 (70.33%)	63 (63.23%)	17 (18.68%)	11 (12.09%)	4–128	4–128	4–128
(n = 91)												
OXA-24	1 (100.00%)	0	0	0	0	1	1	0	0	128	128	64
(n = 1)						(100.00%)	(100.00%)					
OXA-23 & OXA-24	10 (83.33%)	0	2	6	0	6	10	2 (16.67%)	0	32–64	16–64	8–32
(n = 12)						(50.00%)	(83.33%)					
			(16.67%)	(50.00%)								
OXA-23 & OXA-58	2	0	0	1	0	1	1	0	1	64	32	32
									(50.00%)			
(n = 2)	(100.00%)			(50.00%)		(50.00%)	(50.00%)					
**Class B and Class D carbapenemases**												
OXA-23 & NDM	2 (100.00%)	0	0	1	0	1	2	0	0	64–128	32–64	16
(n = 2)				(50.00%)		(50.00%)	(100.00%)					
OXA-24 & NDM	1 (100.00%)	0	0	0	0	1	1	0	0	64	16	16
(n = 1)						(100.00%)	(100.00%)					
OXA-23 & OXA-24 & NDM (n = 1)	1 (100.00%)	0	0	1 (100.00%)	0	0	1	0	0	128	64	32
							(100.00%)					
Carbapenem-sensitive (n = 13)	0	0	13 (100.00%)	0	1	12 (92.31%)	0	0	13 (100.00%)	0.125–2	0.125–1	0.0625–0.5
					(7.69%)							

P, positive; I, Indeterminate; N, negative; n, number of isolates

### The proposed algorithm for early detection of carbapenemase-producing *A*. *baumannii*

In addition to the accuracy of the method, time-consuming and cost-effectiveness are also crucial considerations for the early detection of carbapenemase-producing *A*. *baumannii*, particularly in low-to middle-income countries. A comparison of the sensitivity, time consumption, and extra-consumable equipment of each test method is presented in Table 3. For sensitivity, the CarbAcineto NP test successfully detected carbapenemases as a single screening test (89.09%). However, sCIM was approximately 5.3 times cheaper than the CarbAcineto NP test, although a longer detection time was required for sCIM. The overall highest sensitivity of 98.18% was achieved with the combination of sCIM and CarbAcineto test. The sensitivity of the combination test was higher when positive and indeterminate results were included in the interpretation of the primary sCIM test. Thereafter, the CarbAcineto NP test could be applied only to negative sCIM isolates. The cost for this combination test was lower than that of the CarbAcineto NP test alone; however, a higher sensitivity was achieved.

**Table 3:** Comparison of phenotypic methods for early detection of carbapenemase-producing *A*. *baumannii*.

Detection method	Sensitivity	Time-consuming	Extra-consumable equipments* (approximate price per 100 tests)
CarbAcineto Test^b^	89.09%	2 h	20 ml 5M NaCl200
			microcentrifuge tubes
			20 ml revealing solution
			60 mg imipenem^a^
			(70 USD)
sCIM	71.82%	18–20 h	25 MHA plates
			100 imipenem discs
			10 ml NSS
			(13 USD)
mCIM	32.73%	22–24 h	25 MHA plates
			200 ml TSB
			10 ml NSS
			100 meropenem discs
			100 culture tubes
			(27 USD)
sCIM (positive) + CarbAcineto	96.36%	20–22 h	25 MHA plates
			100 imipenem discs
			10 ml NSS
			6 ml 5M NaCl
			60 microcentrifuge tubes
			6 ml revealing solution
			18 mg imipenem^a^
			(34 USD)
sCIM (positive + indeterminate) + CarbAcineto	98.18%	20–22 h	25 MHA plates
			100 imipenem discs
			10 ml NSS
			2.4 ml 5M NaCl
			24 microcentrifuge tubes
			2.4 ml revealing solution
			7.2 mg imipenem^a^
			(21.4 USD)

* Based on the researcher lab.

^a^ Pharmaceutical Secondary Standard; Certified Reference Material (Sigma-Aldrich, USA)

^b^ All solutions in the reaction were prepared in the research lab according to Dortet L, et al. [17].

## Discussion

According to Ambler’s classification, carbapenemases can be divided into four classes: A, B, C, and D. Class A, C, and D beta-lactamases are serine-based carbapenemases, whereas class B is a group of metallo-beta-lactamases. Carbapenemases reported worldwide are KPC, VIM, IMP, NDM, and OXA types [24, 25]. In *Acinetobacter* spp., most carbapenemases are OXA-type carbapenemases belonging to class D (group 2df) beta-lactamases, which respond varyingly to each beta-lactamase inhibitor [2]. OXA-51, encoded by a gene located on the chromosome of *A*. *baumannii*, was found in this strain as an intrinsic enzyme marker. Although this enzyme belongs to class D beta-lactamases, carbapenemase activity is mostly weak [26]. The insertion of IS*AbaI* may overexpress the enzyme at the 5’ end of OXA-51-like genes [27]. In contrast to OXA-51, OXA-23-like carbapenemases, which were found on the plasmid and within transposons, rendered them transferable between organisms, even cross-species. To date, the *bla*oxa-23-like gene, the first OXA-type β-lactamase gene to be identified from CR-AB, remains the most prevalent globally. Moreover, OXA-23-like has been reported as a dominant transferable OXA-type in Thailand at approximately 68.31% [11]. Enzymatic characterization of OXA-23 showed that it possessed higher hydrolyzing activity against carbapenems than cephalosporins [28]. In addition, the prevalence of *bla*oxa-40/24-like and *bla*oxa-58-like gene occurrences in Thai CR-AB isolates was only 4.92% and 1.09%, respectively [11].

Carbapenems are considered the last resort for infections caused by multidrug-resistant gram-negative bacteria; however, carbapenem resistance is increasingly common in *A*. *baumannii*. Therefore, early and accurate detection of carbapenemase-producing *A*. *baumannii* is urgently needed to achieve the therapeutic efficacy of such infections. Before introducing mCIM, other paper-based detection methods were the modified Hodge test (MHT) and combined disk (CD) tests. MHT was recommended as a carbapenemase detection method in the CLSI guideline until 2017 when it failed to detect MBLs or gave false-positive results for bacteria with complex ESBL combined with porin loss [18, 29]. *A*. *baumannii*, particularly drug resistant-*A baumannii*, is an organism that possesses many resistance mechanisms, including antimicrobial-inactivating enzymes, efflux pumps, and decreased target access. Therefore, MHT is not appropriate for drug resistant*-A*. *baumannii* detection. Therefore, the combined disk (CD) was recommended by the EUCAST guidelines as a carbapenemase detection test [12]. The CD test used the concept of enzyme inhibitors, which could be metal ion chelators such as ethylenediaminetetraacetic acid (EDTA) for class B carbapenemase detection or beta-lactamase inhibitors such as boronic acid for class A and C carbapenemase detection. However, enzyme inhibitors for class D carbapenemases are not currently available [12]. Therefore, the CD test was not appropriate for the detection of *Acinetobacter* spp. because most carbapenemases in *A*. *baumannii* are mainly class D carbapenemases.

Later, the CLSI guideline-recommended mCIM was applied to Enterobacterales and *P*. *aeruginosa* in 2018 [18]. However, mCIM is not recommended for *A*. *baumannii* infections. The mCIM method appears unfavorable for carbapenemase-producing *A*. *baumannii*, as previously demonstrated in a multisite evaluation study [30]. As also observed in our study, mCIM showed lower than 40% sensitivity to detect carbapenemase-producing *A*. *baumannii*. Many attempts have been made to increase the sensitivity and specificity of this test for *A*. *baumannii* by applying additional reagents such as Tris-HCl and Triton-X [31, 32]. sCIM was proposed in 2018 with a procedure adapted from mCIM, which exhibited promising results for *A*. *baumannii* detection with 100% accuracy [19]. In our study, sCIM demonstrated higher sensitivity than mCIM, with 78.86% accuracy. Interestingly, unlike other tests, sCIM used an imipenem disk instead of a meropenem disk, which might help increase the sensitivity of this paper-based detection method [33].

The Carba NP test for *A*. *baumannii* was recommended in the CLSI guidelines during 2015–2017 [13, 18]. The CarbAcineto NP test was then modified from the Carba NP test, but this method was not suggested by the CLSI. Both methods provided results within two h, which was the best advantage of this test. However, the interpretation might be subjective, particularly in a test that gave a slight color change appearance. Moreover, the CarbAcineto NP test provided only approximately 90% sensitivity, similar to the results of our study [34]. It was demonstrated that sCIM and mCIM were less subjective interpretations than the CarbAcineto NP test because the interpretation criteria depended on the size of the inhibition zone.

**Fig 1:**
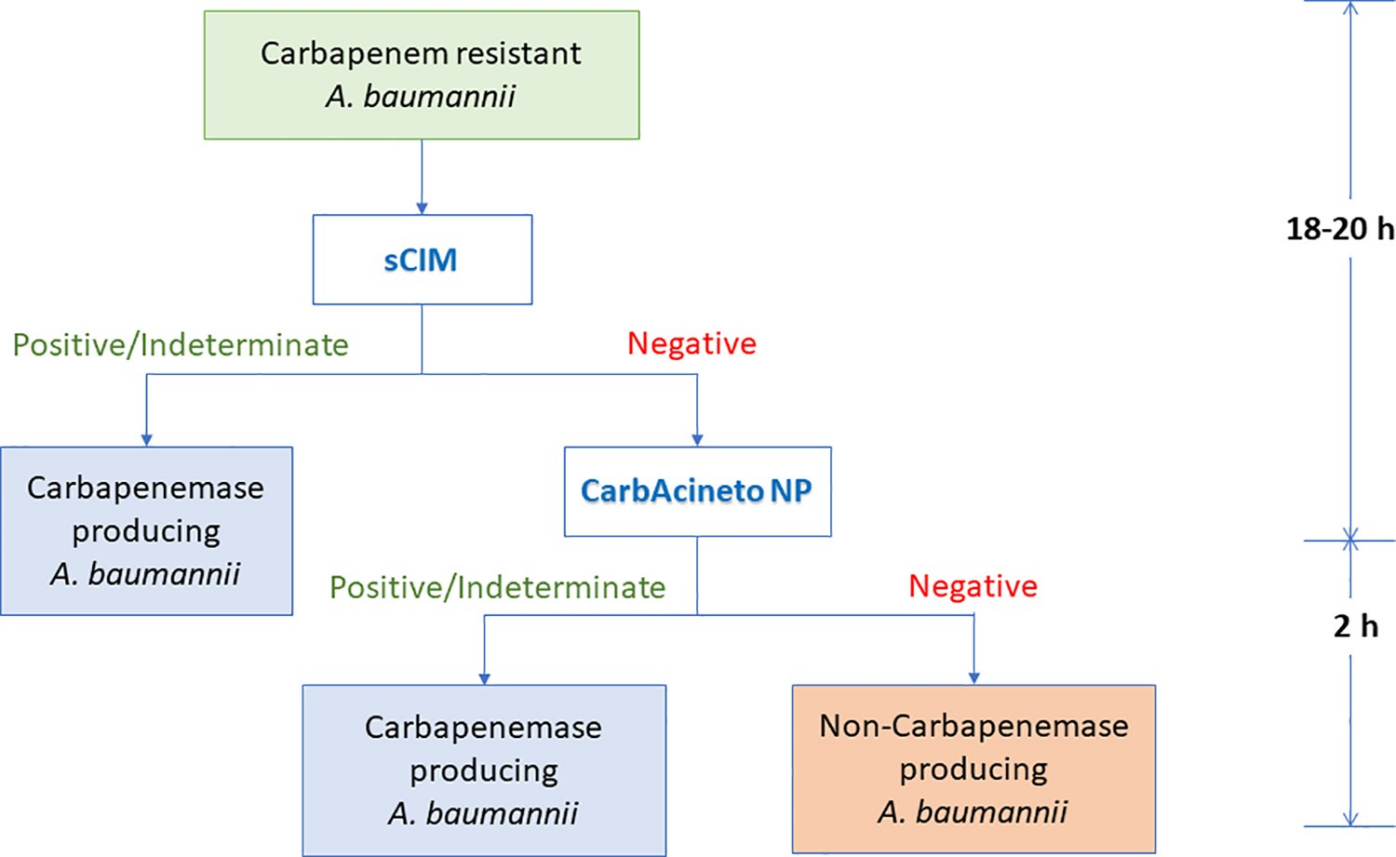
The proposed algorithm for early detection of carbapenemase-producing *A*. *baumannii* for resource-limited settings.

At present, the standard method of phenotypic detection of carbapenemase for *A*. *baumannii* is currently unavailable. In this study, three carbapenemase detection methods, namely, CarbAcineto NP test, modified carbapenem inactivation method (mCIM), and simplified carbapenem inactivation method (sCIM) were evaluated the performance for phenotypic detection of clinically isolated *A*. *baumannii*. Based on the findings of this study, an algorithm for the early detection of carbapenemase-producing *A*. *baumannii* is proposed for resource-limited settings, which can be applied in routine laboratory screening (Fig 1). The proposed combination scheme of the sCIM and CarbAcineto NP test provides up to 98.19% sensitivity. The procedure was first performed with sCIM to primarily screen for carbapenemase-producing *A*. *baumannii*. Then, if negative, the CarbAcineto NP test can be applied as the second confirmatory test. With this proposed scheme, the results can be accurately determined within 20–22 hours. The budget of the test is more affordable and suitable for low to middle-income countries. This study can be helpful for early detection of carbapenem resistance and their transmission when dealing with high-resistant burden and resource-limited areas.

## Supporting information

S1 Table Overall performance of all *A. baumannii* isolates for phenotypic methods for the detection of carbapenemase production and antimicrobial sensitivity tests. (DOCX)
