# Supplementary material for: Comparative study of phenotypic-based detection assays for carbapenemase-producing Acinetobacter baumannii with a proposed algorithm in resource-limited settings
Source: PLoS One. 2021 Nov 4;16(11):e0259686. doi: 10.1371/journal.pone.0259686 (PMC8568097; doi:10.1371/journal.pone.0259686)
Supplement: S1 Table — (DOCX) [file pone.0259686.s001.docx]

**Supporting information**

**S1 Table. Overall performance of all *A. baumannii* isolates for** **phenotypic methods for the detection of carbapenemase production and antimicrobial sensitivity tests.**

| **Sample code** | **Interpretation result** | | | **Carbapenemase gene** | **Minimum Inhibitory Concentration (µg/ml)** | | |
| --- | --- | --- | --- | --- | --- | --- | --- |
|  | **mCIM** | **CarbAcineto NP** | **sCIM** |  | **Imipenem** | **Meropenem** | **Doripenem** |
| 0145 | N | P | P | OXA-23 | 32 | 32 | 16 |
| 0147 | N | P | P | OXA-23 | 64 | 32 | 32 |
| 0148 | N | N | I | OXA-23 | 64 | 16 | 16 |
| 0150 | N | P | P | OXA-23 | 32 | 16 | 16 |
| 0151 | N | P | I | OXA-23 | 16 | 16 | 8 |
| 0153 | N | P | P | OXA-23 | 32 | 16 | 16 |
| 0154 | P | P | P | OXA-23 | 64 | 64 | 16 |
| 0155 | N | N | P | OXA-23 | 32 | 16 | 8 |
| 0156 | N | N | P | OXA-23 | 16 | 8 | 8 |
| 0158 | N | P | P | OXA-23 | 64 | 32 | 16 |
| 0159 | N | N | P | OXA-23 | 32 | 32 | 8 |
| 0163 | N | N | P | OXA-23 | 32 | 32 | 16 |
| 01108 | N | P | I | OXA-23 | 128 | 32 | 8 |
| 01110 | N | P | P | OXA-23 | 64 | 64 | 32 |
| 01111 | N | P | P | OXA-23 | 128 | 64 | 32 |
| 0405 | P | P | P | OXA-23 | 64 | 64 | 32 |
| 0406 | N | P | N | OXA-23 | 32 | 32 | 16 |
| 0408 | P | N | N | OXA-23 | 64 | 32 | 16 |
| 0409 | N | P | I | OXA-23 | 64 | 64 | 32 |
| 0411 | N | P | N | OXA-23 | 32 | 32 | 16 |
| 0412 | P | P | I | OXA-23 | 16 | 8 | 8 |
| 0413 | P | P | N | OXA-23 | 32 | 32 | 16 |
| 0415 | P | P | P | OXA-23 | 16 | 8 | 4 |
| 0416 | N | P | N | OXA-23 | 64 | 64 | 32 |
| 0420 | N | P | P | OXA-23 | 64 | 32 | 16 |
| 0422 | N | P | P | OXA-23 | 64 | 32 | 16 |
| 0424 | N | P | P | OXA-23 | 64 | 32 | 16 |
| 0427 | P | P | P | OXA-23 | 64 | 32 | 16 |
| 0428 | N | P | P | OXA-23 | 64 | 32 | 16 |
| 0430 | N | P | N | OXA-23 | 64 | 32 | 16 |
| 0501 | N | P | P | OXA-23 | 64 | 64 | 64 |
| 0503 | N | P | P | OXA-23 | 32 | 32 | 16 |
| 0507 | N | P | N | OXA-23 | 32 | 32 | 16 |
| 0508 | N | P | I | OXA-23 | 64 | 64 | 32 |
| 0511 | N | P | P | OXA-23 | 64 | 32 | 16 |
| 0520 | N | P | P | OXA-23 | 32 | 32 | 16 |
| 0602 | N | P | N | OXA-23 | 32 | 32 | 16 |
| 0603 | N | P | I | OXA-23 | 128 | 64 | 64 |
| 0606 | N | P | P | OXA-23 | 32 | 32 | 16 |
| 0608 | N | n | I | OXA-23 | 64 | 32 | 32 |
| 0609 | N | P | P | OXA-23 | 64 | 32 | 16 |
| 0610 | N | P | P | OXA-23 | 32 | 32 | 16 |
| 0612 | N | P | P | OXA-23 | 32 | 32 | 16 |
| 0614 | N | P | P | OXA-23 | 64 | 64 | 32 |
| 0615 | N | P | P | OXA-23 | 128 | 64 | 32 |
| 0617 | N | P | I | OXA-23 | 64 | 64 | 32 |
| 0619 | N | P | P | OXA-23 | 64 | 64 | 32 |
| 0620 | N | P | P | OXA-23 | 32 | 32 | 32 |
| 0623 | N | P | P | OXA-23 | 32 | 32 | 16 |
| 0627 | N | P | I | OXA-23 | 32 | 128 | 32 |
| 0703 | P | P | P | OXA-23 | 4 | 4 | 4 |
| 0710 | N | P | P | OXA-23 | 64 | 64 | 16 |
| 0712 | P | P | P | OXA-23 | 64 | 64 | 32 |
| 0802 | N | P | I | OXA-23 | 16 | 16 | 8 |
| 0807 | N | P | N | OXA-23 | 32 | 32 | 32 |
| 0808 | N | P | P | OXA-23 | 64 | 64 | 64 |
| 0810 | N | P | P | OXA-23 | 64 | 64 | 64 |
| 0811 | N | P | P | OXA-23 | 32 | 32 | 32 |
| 0815 | N | N | N | OXA-23 | 64 | 64 | 32 |
| 0816 | N | P | I | OXA-23 | 64 | 64 | 32 |
| 0818 | N | P | I | OXA-23 | 32 | 32 | 16 |
| 0905 | N | P | P | OXA-23 | 32 | 16 | 8 |
| 0907 | N | P | N | OXA-23 | 128 | 32 | 16 |
| 0921 | N | P | P | OXA-23 | 64 | 32 | 16 |
| 0924 | P | P | P | OXA-23 | 32 | 16 | 8 |
| 1102 | N | P | P | OXA-23 | 8 | 8 | 4 |
| 1104 | P | N | P | OXA-23 | 16 | 8 | 8 |
| 1106 | N | P | P | OXA-23 | 64 | 32 | 32 |
| 1107 | P | P | P | OXA-23 | 64 | 32 | 16 |
| 1109 | P | P | P | OXA-23 | 16 | 8 | 8 |
| 1111 | P | P | P | OXA-23 | 16 | 8 | 4 |
| 1112 | N | P | P | OXA-23 | 32 | 32 | 16 |
| 1113 | P | P | P | OXA-23 | 32 | 32 | 16 |
| 1114 | P | P | P | OXA-23 | 16 | 8 | 8 |
| 1115 | P | P | P | OXA-23 | 32 | 16 | 8 |
| 1117 | P | P | P | OXA-23 | 32 | 32 | 16 |
| 1118 | P | N | P | OXA-23 | 32 | 16 | 16 |
| 1119 | P | P | P | OXA-23 | 128 | 128 | 64 |
| 1120 | P | P | P | OXA-23 | 16 | 16 | 8 |
| 1121 | P | P | P | OXA-23 | 64 | 64 | 32 |
| 1122 | P | P | I | OXA-23 | 16 | 8 | 4 |
| 1123 | P | P | P | OXA-23 | 16 | 8 | 4 |
| 1124 | P | P | I | OXA-23 | 16 | 16 | 8 |
| 1125 | N | P | P | OXA-23 | 64 | 32 | 32 |
| 1127 | P | P | I | OXA-23 | 16 | 16 | 8 |
| 1128 | P | P | P | OXA-23 | 16 | 8 | 16 |
| 1130 | N | P | P | OXA-23 | 128 | 128 | 128 |
| 1305 | N | P | P | OXA-23 | 32 | 32 | 32 |
| 1306 | N | P | P | OXA-23 | 32 | 16 | 32 |
| 1310 | N | P | P | OXA-23 | 32 | 64 | 32 |
| 1322 | N | P | I | OXA-23 | 32 | 32 | 16 |
| 0716 | N | P | P | OXA-23, NDM | 64 | 32 | 16 |
| 0719 | P | P | P | OXA-23, NDM | 128 | 64 | 16 |
| 0706 | N | P | P | OXA-24 | 128 | 128 | 64 |
| 0709 | N | P | P | OXA-24, NDM | 64 | 16 | 16 |
| 0181 | N | N | P | OXA-23, OXA-24 | 32 | 16 | 16 |
| 0429 | P | P | P | OXA-23, OXA-24 | 32 | 16 | 16 |
| 0701 | N | P | I | OXA-23, OXA-24 | 64 | 32 | 16 |
| 0702 | N | P | P | OXA-23, OXA-24 | 32 | 32 | 8 |
| 0705 | N | P | P | OXA-23, OXA-24 | 64 | 64 | 16 |
| 0707 | N | P | P | OXA-23, OXA-24 | 64 | 32 | 16 |
| 0708 | N | N | P | OXA-23, OXA-24 | 32 | 32 | 16 |
| 0714 | P | P | I | OXA-23, OXA-24 | 64 | 64 | 32 |
| 1318 | P | P | P | OXA-23, OXA-24 | 64 | 32 | 32 |
| 1320 | P | P | P | OXA-23, OXA-24 | 32 | 32 | 16 |
| 1330 | P | P | P | OXA-23, OXA-24 | 64 | 32 | 32 |
| 1331 | P | P | P | OXA-23, OXA-24 | 32 | 32 | 16 |
| 0718 | P | P | P | OXA-23, OXA-24, NDM | 128 | 64 | 32 |
| 0404 | P | P | P | OXA-23, OXA-58 | 64 | 32 | 32 |
| 0418 | N | P | N | OXA-23, OXA-58 | 64 | 32 | 32 |
| 0532 | N | N | N | ND | 0.125 | 0.125 | 0.0625 |
| 0613 | I | N | N | ND | 0.5 | 0.5 | 0.125 |
| 0218 | N | N | N | ND | 0.5 | 0.50 | 0.125 |
| 0704 | N | N | N | ND | 2 | 1 | 0.5 |
| 0417 | N | N | N | ND | 1 | 1 | 0.125 |
| 0410 | N | N | N | ND | 1 | 1 | 0.125 |
| 0920 | N | N | N | ND | 0.25 | 0.125 | 0.25 |
| 0407 | N | N | N | ND | 1 | 1 | 0.25 |
| 1101 | N | N | N | ND | 0.25 | 1 | 0.25 |
| 0720 | N | N | N | ND | 1 | 1 | 0.25 |
| 0711 | N | N | N | ND | 1 | 1 | 0.125 |
| 0419 | N | N | N | ND | 2 | 1 | 0.125 |
| ATCC | N | N | N | ND | 0.5 | 0.5 | 0.125 |

N: negative; P: positive; I: Indeterminate; ND: not detectable
